# Why do we need a…Journal?

**DOI:** 10.4103/0972-0707.53333

**Published:** 2009

**Authors:** Velayutham Gopikrishna

**Affiliations:** Department of Conservative Dentistry, Meenakshi Ammal Dental College, Alapakkam Main Road, Maduravoyal, Chennai - 600 095, Tamil Nadu, India E-mail: editor@jcd.org.in

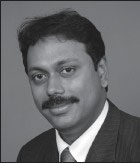



Perhaps it is a sad commentary that both in medicine and dentistry, most of the information today is conveyed through the spoken media, in particular, the continuing education circuit. Nonetheless, this is understandable because the life of an average professional has become both busy and too good to take undivided time to read, learn, and critically think.

To do a job well requires skills and knowledge. Dental education followed by postgraduate training provides the clinician with a set of very useful skills. With the improvements in endodontic instruments, however, clinicians' manual skills might have become less critical. But how about knowledge? Is it possible for any clinician to rigorously test the new materials in the market and reach a conclusion on whether to adopt the latest innovation or not? A good clinician is the one who betters himself/herself continually by testing his/her skills constantly, increases his/her knowledge of the tissues he/she is affecting, assesses the outcomes of his/her treatment, and is keen to deliver the form of treatment that meets the patient's desires best. Said differently, a true clinician seeks wisdom and truth in himself/herself and in what is around. This is exactly where a journal helps and supplements a clinician's quest for perfection.

Journals separate truth from a collection of facts, as do the academic establishments with their curricula. In the world around, oftentimes, what one sees is not what it is – such is the case in a magic show. For the most part, as it is entertainment and is harmless, one chooses to be amazed rather than to go to the backstage and discover how it is really done. In matters of our profession, however, we seek the truth, and more significantly, we want to seek it ourselves. Instinctively, we do not believe everything we hear, certainly not before we had a chance to test it against our standards of truth. This is a form of peer-review process. Talking is cheap; anyone can say anything he/she wants in a society where free speech is guaranteed by the constitution. But a speech (lecture) is meaningless if the proof is lacking. Newspapers too, in the absence of supporting data in their columns, become tabloids. Proof, accreditation standards (for academic institutions), substance, truth, and the like, are the building blocks of better knowledge.

In contrast, journals print only what is judged to be truthful. Journal articles are reports of scientific studies conducted with rigorous standards as judged through the peer-review process. Unlike a textbook, journals present the truth while review papers or textbooks express the opinions of their authors. In contrast, journals that publish the breakthrough information originating from experimental research data are the instruments of advancement.

The growth of any profession is based not only on the amount of research and publications on materials and techniques but also, more importantly, on the proportion of fellow professionals who READ these publications and put them into practice! Knowledge can be researched, published, and shared but knowledge which is not read and imbibed is useless. Hence, the philosophy of “publish or perish” has to go hand in hand with the philosophy of “read or become redundant.”

The endeavor of the Journal of Conservative Dentistry is not only to provide a platform for the dissemination of knowledge being generated in our field across the country but also to be the harbinger of change and betterment in clinical practice amongst professionals.

Keep reading….

